# Stereotactic Radiosurgery Hypophysectomy for Palliative Treatment of Refractory Cancer Pain: A Historical Review and Update

**DOI:** 10.3389/fonc.2020.572557

**Published:** 2020-12-17

**Authors:** M. Benjamin Larkin, Patrick J. Karas, John P. McGinnis, Ian E. McCutcheon, Ashwin Viswanathan

**Affiliations:** ^1^Department of Neurosurgery, Baylor College of Medicine, Houston, TX, United States; ^2^Department of Neurosurgery, University of Texas, MD Anderson, Houston, TX, United States

**Keywords:** cancer pain, hypophysectomy, hypothalamus, malignancy, palliative care, radiation oncology, stereotactic radiosurgery, SRS

## Abstract

Medically refractory pain in those with advanced cancer significantly reduces one’s quality of life. Therefore, palliative interventions to mitigate cancer pain and reduce opioid requirements are necessary to reduce patient suffering and opioid-induced side effects. Hypophysectomy, a largely forgotten pain procedure with several technical variations, has been repeatedly studied in small series with encouraging results, though historically has been fraught with complications. As a result, the minimally invasive and more tolerable stereotactic radiosurgery (SRS) hypophysectomy has resurfaced as a possible treatment for cancer-related pain. While the mechanism of pain relief is not entirely understood, the hypothalamohypophyseal axis appears to play an essential role in pain perception and transmission and involves C fiber signal processing and downstream modulation of the brainstem and spinal cord *via* the hypothalamus. This review highlights the role of hypophysectomy in alleviating advanced cancer pain, both in hormonal and nonhormonal malignancy and the current mechanistic understanding of pain relief for the three primary hypophysectomy modalities used historically: surgical and chemical adenolysis, as well as the more recent, SRS hypophysectomy. Given the lack of high-quality evidence for stereotactic radiosurgery hypophysectomy, there is a need for further rigorous and prospective clinical studies despite its ideal and noninvasive approach.

## Introduction

A significant subset of patients with advanced cancer suffer from refractory pain. As supported by World Health Organization guidelines, aggressive opioid management has been shown to control pain in 70% of cancer patients, with 30% of patients continuing to have refractory pain (1).

Many therapeutic options exist for the treatment of patients with refractory cancer pain. Surgical treatments include intrathecal drug delivery, ablative techniques including myelotomy, cordotomy, and cingulotomy, and neuromodulation in cancer survivors. Alternative and complementary treatments focusing on the psychological aspects of chronic pain, such as cognitive-behavioral therapy, are also essential for effective long-term pain management.

In the setting of a rising opioid epidemic, addiction concerns are leading to under-treatment of cancer pain due to the inability to obtain opioid medications among cancer patients under increasingly tight regulatory dispenser rules (2, 3). In order to achieve effective palliation and improve quality of life, the prescriber must balance therapeutic efficacy with often concomitant opioid-induced neurotoxicity. The treatment of cancer-related pain remains a considerable challenge.

Hypophysectomy is a largely forgotten palliative pain procedure in the modern era. Historically, it was used for the palliative treatment of refractory pain in patients with hormone-sensitive cancer, with particular efficacy for diffuse bony metastatic pain. With technological improvements in stereotactic radiosurgery, radiosurgery ablation of the pituitary has resurfaced as a minimally invasive tool to provide pain relief.

In this review, the authors focus on the history of hypophysectomy for pain and the different pain pathways, which may provide an understanding of the pain relief mechanisms following hypophysectomy, as well as a review of hypophysectomy outcomes. While further clinical trials are needed, radiosurgery hypophysectomy may be a promising approach for palliative pain relief in patients who are refractory to other treatments.

### History

In 1953, Luft & Olivecrona were the first to describe pain relief associated with secondary prostate and breast cancer lesions following hypophysectomy for tumor control (4). Transcranial approaches were abandoned as transnasal transsphenoidal approaches, and the use of a stereotactic frame became widely favored in the 1970s for pituitary neuroadenolysis (NALP). This included radiotherapy, ultrasound, cryoablation, thermocoagulation, interstitial radiation, and alcohol instillation (5). First described by Greco et al., but popularized by Moricca, alcohol-induced NALP became the most widely used over this period and led to complete pain relief in 70-80% of patients, allowing as many as 50% of patients to cease opioid therapy (5–7). The overall clinical results for complete pain relief from cancer pain for both surgical and chemical hypophysectomy among 1101 patients was 64.4% (8) (**Tables 1** and **2**).

**Table 1:** Surgical hypophysectomy.

Study	Number of Patients (N)	Procedure	Pain Results	Side Effects (N)
Forrest et al., 1959 (9)	45	Radioactive Implantation of Pituitary	Pain not assessed	Vision loss (8/45); CN3 palsy (2/45); DI, CSF leak (2/45);
Scott et al., 1962 (10)	17	R frontal hypophysectomy	6/10 with subjective improvement	No mention of side effects
Kapur et al., 1969 (11)	63	Transphenoidal Hypophysectomy	No quantification of pain improvement	Hemorrhage (1/63), CSF leak (4/63), meningitis (1), cortisol deficiency (3), DI (11), infection (1)
Zervas et al., 1969 (12)	164, 66 for breast cancer	Radiofrequency ablation	31% (of the 66) with pain improvement (without remission)	CSF leak, meningitis
Hardy et al., 1971 (13)	All technique, no results	Transphenoidal Hypophysectomy	-	–
Maddy et al., 1971 (14)	20	Cryohypophysectomy	13 patients with subjective improvement	CSF (2), transient DI (7), transient SIADH (5)
Thompson et al., 1974 (15)	47	Transcranial Hypophysectomy	28 (60%) pain improvement (1–3 mo)	Cerebral edema or hematoma causing death (4/60), CSF leak (1), monocular vision loss (1)
Gros et al., 1975 (16)	128	Transphenoidal Hypophysectomy	85 with pain improvement	CSF leak (26), DI (71), meningitis (2)
Tindall et al., 1977 (17)	44 (subgroup of Tindall 1979)	Transphenoidal Hypophysectomy	13/17 and 19/23 with pain improvement	CSF leak (7) meningitis (2), DI (39)
Silverberg et al., 1977 (18)	17	Transphenoidal Hypophysectomy	12 had subjective improvement, mean 3.8 mo	CSF leak (1), DI (37%)
Tindall et al., 1979 (19)	53	Transphenoidal Hypophysectomy	39/43 with pain relief, mean relief 2.2 mo	Death-carotid injury (1), DI (40), CSF leak (6)
West et al., 1979* (20)	19	Cryohypophysectomy	-	–
Takeda et al., 1983 (21)	18, 17 with pain	Transphenoidal Hypophysectomy	15/17 improvement in pain	CSF leak (4), meningitis (2), hemorrhage/death (1)

*Full text article not available.

**Table 2:** Chemical hypophysectomy.

Study	Number of Patients (N)	Procedure	Pain Results	Side Effects (N)
Moricca et al., 1976* (22)	–	Alcohol adenolysis	–	–
Corssen et al., 1977 (23)	24	Alcohol adenolysis	13 complete + 10 improvement	Ophthalmoplegia (1), nosebleed (1)
Lipton et al., 1978 (24)	92	Alcohol adenolysis	38 complete relief (median, 4 mo); 28 partial, 26 none	Death (4), DI, hemorrhage, CSF leak, III palsy transient, empyema, hypothalamic injury,
Madrid et al., 1979* (25)	–	Alcohol adenolysis	–	–
Miles et al., 1979* (26)	–	Alcohol adenolysis	–	–
Levin et al., 1980 (27)	17 (29 all cancers)	Alcohol adenolysis	Pain improvement 16/17 and 11/12	CSF leak (1), III palsy (4), visual field deficit (3), DI (all with pain improvement)
Williams et al., 1980 (28)	11	Alcohol adenolysis	8/11 ‘significant’ pain improvement	Diabetes Insipidus (2)
Takeda et al., 1983 (29)	102	Alcohol adenolysis	Pain relief 82/102	Temporary headaches (50), transient hyperthermia (‘few’), hallucinations (‘few’); visual defects (10), meningitis (2), transient ophthalmoplegis (4),

*Full text article not available.

Despite promising pain relief outcomes, early reports of NALP were associated with significant morbidity, including diabetes insipidus (75%), rhinorrhea (20%), hypopituitarism requiring replacement therapy (15%), and decreased libido (5, 7, 23). Rare and severe complications include cerebral spinal fluid leak, infections (*e.g.*, meningitis), visual dysfunction, intrasellar hemorrhage, and death (5, 8). NALP for cancer pain fell out of favor because of this high morbidity.

Stereotactic radiosurgery was first used to treat malignancy-related refractory pain in 1968, targeting the centromedian thalamic nucleus (30). Eventually, other terminal thalamic endpoints for the paleospinothalamic tract fibers also became radiosurgery targets for non-malignant refractory pain. These included the mediodorsal, centromedian, intralaminar, and parafascicular nuclei (31–36).

However, for refractory malignant pain, stereotactic radiosurgical ablation of the pituitary is superior to thalamic ablation (37). Ablative stereotactic radiosurgery of the pituitary was first described by Backlund et al. in 1972 but not further investigated for three decades (8, 38, 39). These studies suggest that stereotactic radiosurgery (SRS) is more efficacious and safer than hypophysectomy. Older studies with 200 to 250 Gy doses had similar efficacy to more modern series with improved MRI and CT imaging that used 160 Gy. Additionally, there are significantly fewer side effects over 24 months following SRS in patients with cancer pain (**Table 3**) (38). Hayashi et al. have since utilized SRS for treatment of both cancer pain and chronic neuropathic thalamic stroke pain (8, 41, 42). Patients with malignancy-related pain had significantly higher rates of sustained pain reduction than those with non-malignancy related pain (8, 37, 39, 40, 42).

**Table 3:** Stereotactic radiosurgery hypophysectomy.

Study	Number of Patients (N)	Method of Delivery	Pain Results	Side Effects (N)	Max dose (krad/Gy)
Backlund et al., 1972 (38)	8	2-3 isocenters, 3 × 5 or 3 mm × 7 mm cross-sectional beams	4/4 survivors ‘relief of pain’	Hormonal deficiencies (8)	20 or 25 krad
Hayashi et al., 2002 (8)	9	1 isocenter, 8 mm × 4 mm patients; 2 isocenters, 4 mm × 5 mm patients	9 pain free (permanently)	None	150–200 Gy
Hayashi et al., 2003 (40)	6	1 isocenter, 8 mm collimator	6 pain free (1–4 mo)	None	160 Gy
Kwon et al., 2004 (39)	7	1 isocenter 8 mm × 3 mm patients; 2 isocenters 4 mm × 4 mm patients	7 pain reduction, 5 without relapse	DI/hypopituitarism (1)	150–160 Gy

### Current Research in SRS Hypophysectomy

For more than a decade, there has been little discussion of hypophysectomy for the treatment of cancer pain. Since Hayashi et al. last published their original series in 2002, there have only been two further published studies (39, 43). The rise of intrathecal opioid administration, though not without significant complication (i.e., infection, neurotoxicity, endocrinopathies, and device malfunction) has largely driven hypophysectomy for the treatment of refractory cancer pain out of favor (44, 45).

Radiosurgery hypophysectomy studies to date have been small, non-randomized, and prospective trials providing low-level evidence to support its use (8, 38, 39, 43). Some include limited descriptions of the types of pain treated, lack well-validated pain scale utilization, and report limited endocrine and pain control follow-up. However, two clinical trials are underway investigating the safety and efficacy of single fraction radiosurgery hypophysectomy in reducing cancer pain from bone metastases and opioid-refractory pain in palliative care patients (46, 47).

**Figure 1:**
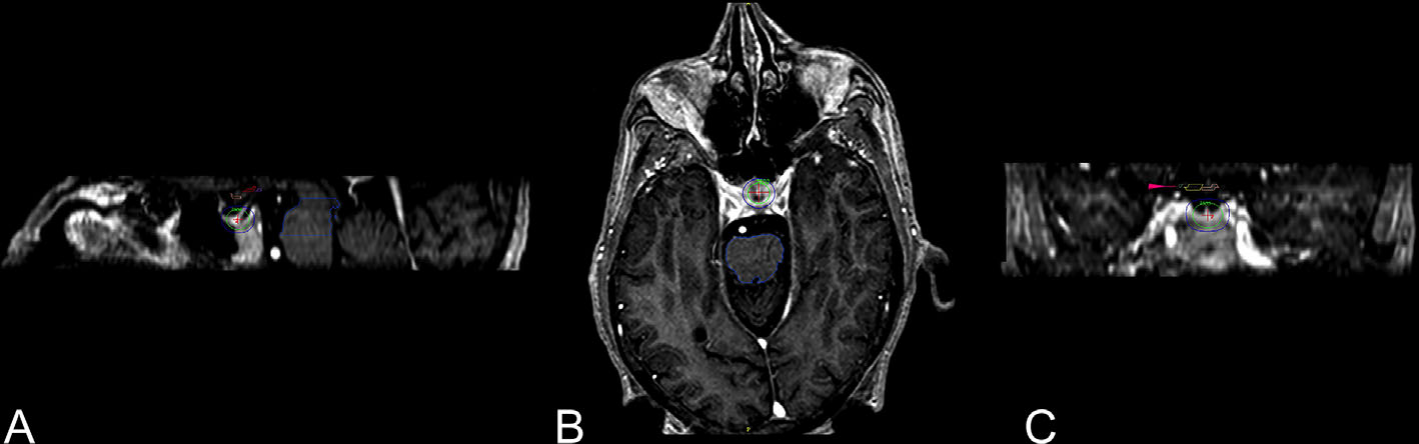
Three-dimensional views of 8-mm collimator shot to the neurohypophysis (43). Three-dimensional views of the dose distribution to the 8-mm collimator shot with higher isodose lines posterior part of the neurohypophysis. **(A)** Sagittal view with the pituitary gland and stalk covered with green 50% isodose line (75 Gy), exterior to it is the blue 25% isodose line (37.5 Gy). **(B)** Axial view. **(C)** Coronal view, visual pathway (small pink arrow).

**Figure 2:**
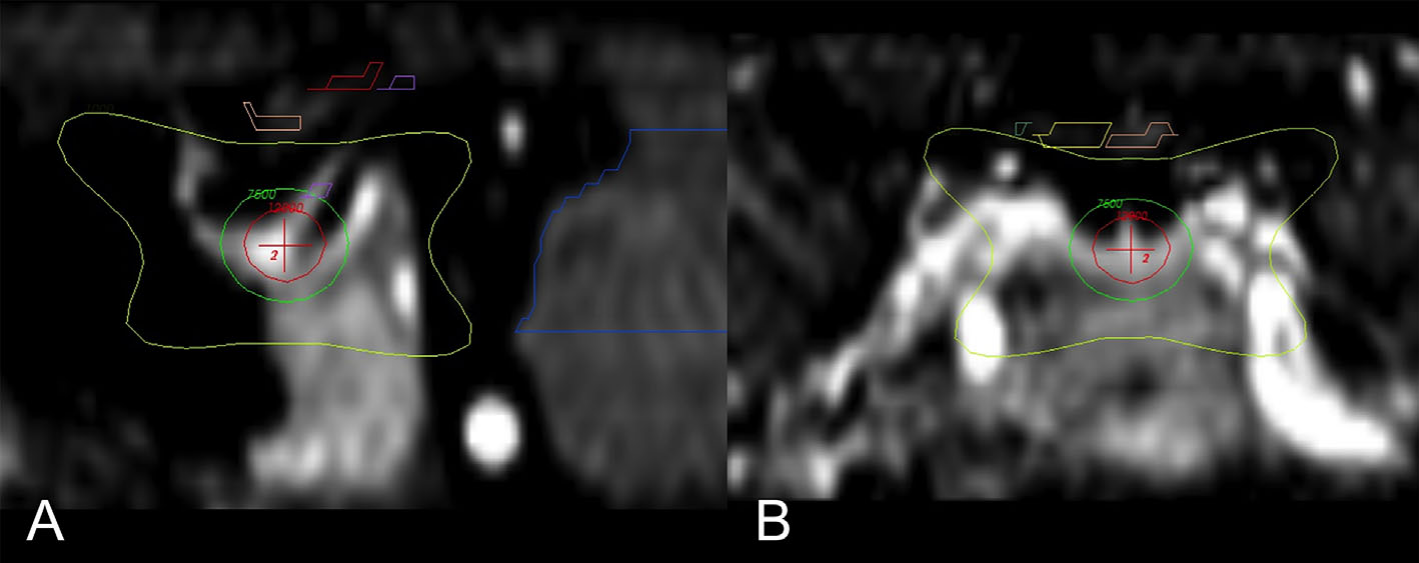
Main sagittal and coronal views of the region of interest and dose distribution (43). **(A)** Enlarged sagittal section of the hypophysis; isocenter of the 8-mm shot (red-cross) is directed to the neurohypophysis. Seen is the 120 Gy (red circle), 75 Gy (green), and 10 Gy (yellow) isodose lines. **(B)** Enlarged coronal section with the 10 Gy (yellow) isodose line; the optic pathway is seen above (yellow and pink).

While the exact mechanism of chemical and/or surgical hypophysectomy remains elusive, it is as well unknown if the pain relief following radiosurgery hypophysectomy is achieved *via* the same set of mechanisms or another. Radiosurgery hypophysectomy has reported success in treating hormonal-related cancer pain, nonhormonal cancer pain without bone metastases, and non-malignant thalamic pain (33, 35, 39, 41, 43, 48). The target center’s location is typically at the junction of the stalk and the superior neurohypophysis where oxytocin is stored. (**Figures 1** and **2**) Similar to theories yet to be discussed, it is thought that SRS hypophysectomy may redirect oxytocin toward hypothalamic regions affecting pain modulation (22, 26, 27, 29, 43, 49). More recent studies provide additional evidence supporting the theory that oxytocin plays a central role in pain modulation (50, 51).

Most importantly, while the rate of adverse events with chemical hypophysectomy prevented its continued use, current evidence demonstrates far fewer adverse events for radiosurgery hypophysectomy. From all available studies regarding the use of radiosurgery for cancer pain, only 8 of 23 patients (35%) had adverse endocrinologic events (*e.g.*, long term hormonal deficiencies, transient diabetes insipidus) (37). When excluding patients with the highest doses of radiation (200-250 Gy), only 1 of 16 patients (6.3%) developed a transient endocrinologic adverse event.

Currently, there is no standard of care treatment for radiosurgery hypophysectomy, and this modality remains investigational. Its use should be limited to the palliative treatment of patients with incurable malignancies and evidence of multiple bone metastases with greater than four weeks life expectancy who have demonstrated intractable nociceptive or mixed pain uncontrolled by opioids, medical management, injections/ablations or surgical and conventional radiation interventions that is limiting the patient’s function and quality of life. Patients who are candidates for this treatment should be thoroughly reviewed/approved by a committee which includes a palliative care specialist, oncologist, pain management anesthesiologist, radiation oncologist, and a neurosurgeon.

### Etiology of Cancer-induced Bone Pain

The causes of pain in cancer are numerous. The tumor can directly invade or distort tissue, impinge and invade nerves, or obstruct hollow viscus and ductal structures leading to pain (49). Pain also arises as a common side effect of radiation, surgery, and chemotherapy that may be essential for comprehensive oncologic treatment (49, 52). Patients frequently have difficulty distinguishing a singular cause.

Metastatic bone pain is the most common complaint among cancer patients and is often poorly controlled (53). Cancer-induced bone pain is a complex entity involving components of both inflammatory and neuropathic pain. The main driver of bony pain may be secondary to compression of periosteal sensory nerve terminals between growing tumors and hard cortical bone compared to other more compliant tissue types (53). Nevertheless, high variability and unpredictability make metastatic bony pain challenging to manage (54). Despite this difficulty, patients with metastatic bone pain responded more favorably to hypophysectomy compared to patients with other etiologies of cancer-related pain (11, 12, 16, 19, 49).

The primary afferent neurons that innervate the periosteum are mostly small C-fibers and Aδ neurons that contain free fiber-endings responsible for sensing noxious stimuli. Additionally, larger Aβ neurons with encapsulated endings are responsible for the sensation of innocuous tactile and kinesthetic stimuli (53). C-fibers are unmyelinated and transmit signals slowly at speeds less than 2 m/sec. This slow transmission is responsible for sensations of deep, burning, “slow pain.” In contrast, thin myelination on Aδ neurons allows for faster transmission at speeds ranging from 2 to 12 m/sec. This faster transmission is responsible for thermal and mechanical nociception, or “fast pain.” The Aβ neurons play a role in pain processing in other tissue types, but their exact role in bone and periosteum pain generation is not clear (52).

The ascending sensory pathways for acute mechanical stimulation of the bone, thought to be activated in diffuse metastatic bony pain, originate in Lamina I of the spinal dorsal horn and ascend in the spinoparabrachial pathway (53, 55, 56). This pathway is distinct from the spinothalamic tract and postsynaptic dorsal column responsible for sensation from the stimulation of cutaneous and visceral structures (53, 55, 56). The spinoparabrachial neurons ascend to the contralateral lateral parabrachial nucleus, which is also involved in the affective and motivational aspect of pain sensation. The parabrachial nucleus then projects to the amygdala, nucleus of the solitary tract, ventrolateral medulla, periaqueductal gray, medial thalamus, and hypothalamus (53, 57, 58). This pathway provides an anatomic substrate for the dual roles of nociceptive and affective pain seen in patients with cancer-induced bone pain.

Cortical evoked potentials have been used as an objective measure of pain to explore how modulation to Aδ and C-fiber transmission influences the perception of painful stimuli (59). Only one study has looked specifically at the cortical evoked potential representation from mechanical bone stimulation (60). As previously discussed, the afferent pain signals for acute cutaneous, visceral, and bone stimulation travel *via* different dorsal spinal cord lamina and afferent pathways. Acute mechanical bone stimulation elicited short latency (<50 ms) evoked potentials in both the primary (SI) and secondary (SII) somatosensory cortices that correlated to the intensity level of the stimulus (53, 60). These signals may be representative of the periosteal stimulation that is associated with breakthrough pain experienced by patients with cancer-induced bone pain, and likely reflective of fast, sharp pain signals from Aδ neurons (53). Cortical responses with longer latency evoked potentials that would be representative of inflammatory C-fiber stimulation were not seen in the primary somatosensory cortex (60).

### Mechanisms for Cancer Pain Relief *via* Hypophysectomy

The mechanism of pain relief after hypophysectomy is not well understood. Unlike cingulotomy or subcaudate frontal leukotomy, hypophysectomy does not primarily produce relief by amelioration of affective components of pain. Early theories hypothesized that reduced pituitary hormones and tumor regression contributed to pain reduction. The observation that prompt pain relief was achieved following oophorectomy, adrenalectomy, or orchiectomy in metastatic breast and prostate further supported this hypothesis. These parallels suggest a common mechanism, such as the disruption of a feedback loop resulting in a hypothalamic response or an increase in a yet unidentified substance that accounted for the observed pain relief (49, 61).

However, this theory did not explain the immediate pain relief that was achieved by many patients. Pain relief after hypophysectomy often precedes any objective tumor remission. Moreover, pain relief persists despite tumor progression and the return of normal pituitary function. Furthermore, patients with nonhormonal tumors also experienced pain relief (27, 40, 49). An alternative hypothesis to explain these observations looked toward precursor molecules (pre-pro-opiomelanocortin) to endogenous brain peptides and endorphins, which have an opioid-like effect, and localized to the pituitary gland and hypothalamus (17, 40). Hypophysectomy was thought to cause increased activity of these precursor molecules with opioid-like downstream effects leading to pain relief.

In 1979, Yanagida et al. investigated the mechanism of pain relief after chemical hypophysectomy by measuring sensory evoked potentials before and after tooth pulp stimulation (Aδ and C-fiber neurons) in primates who had undergone hypophysectomy (61). Tooth pulp-evoked potentials’ (TPEPs) were recorded from the primary somatosensory cortex (PSC), centromedian thalamic nucleus (CM), and the midbrain reticular formation (MRF) following painful stimulation of tooth pulp to determine whether the pain response was reversible with the administration of naloxone (61). If the analgesia obtained following chemical hypophysectomy was mediated entirely *via* an increase in endorphins, naloxone was hypothesized to reverse the effect and undo the pain relief achieved by hypophysectomy (61). TPEP amplitudes, though not latencies, were decreased in all subjects regardless of the extent of pituitary destruction. Naloxone administration reversed only the TPEPs recorded from the PSC but not those recorded from the CM or MRF. This reversal was also only observed in primates who had undergone complete pituitary destruction. The reversal of PSC TPEPs after naloxone administration suggests that endorphins likely played some role in pain relief. However, the lack of changes in CM and MRF responses and differences seen as a result of the extent of pituitary destruction suggests an unidentified explanation for pain relief, such as interference with sensory pathways, as decreased TPEP amplitude was universally demonstrated (61).

Takeda et al. demonstrated that immediately after hypophysectomy, there was a transient sharp rise in CSF β-endorphins. However, the CSF β-endorphins returned to pre-treatment levels by the third postoperative day, suggesting a different mechanism for long term pain relief after hypophysectomy. Several other studies reported the failure of analgesia reversal with naloxone administration following NALP, reinforcing the idea that while endorphins have a role in the immediate pain relief achieved by patients after hypophysectomy, they are not the mechanism by which lasting analgesia is achieved (21, 27, 29, 62).

In contrast to the sharp rise and fall of endorphins in the CSF, Takeda and colleagues also showed that the concentrations of ACTH were markedly increased both in the early postoperative period and at two months following hypophysectomy (29). Furthermore, patients who did not have CSF elevations of ACTH never achieved pain relief. Interestingly, patients with pain relief also had elevated CSF concentrations of both thyrotropin-releasing hormone and vasopressin, which may provide analgesia *via* additional pathways discussed later.

Hypophysectomy may also influence pain fiber pathways differently. Radiant heat dolorimetry and ischemic pain induced *via* tourniquet were used to test for differences in pain thresholds between human patients who did and did not experience complete cancer pain relief following NALP (29). Radiant heat dolorimetry envokes temperature pain *via* stimulation of Aδ and C-fibers. In contrast, tourniquet-induced ischemic pain is mediated mainly by C-fibers. While no difference in pain threshold was detected between patient groups using radiant heat dolorimetry, patients with complete cancer pain relief following NALP had significantly increased resistance to ischemic pain measured with tourniquet time, suggesting NALP may be more effective at relieving pain transmitted *via* C-fibers but not Aδ-fibers (29, 63).

Along these lines, Simpson and colleagues demonstrated that neuronal activation of the lateral anterior hypothalamus was capable of differentially modulating descending control of nociception in A and C-fiber evoked spinal nociception (53, 64). Interestingly, in earlier chemical hypophysectomy attempts, it was noticed that a patient’s sense of pain felt from acute injury was not altered (26, 49, 65). This observation suggests that hypophysectomy may selectively inhibit transmission of inflammatory and/or chronic cancer-related pain *via* C-fibers afferents while still allowing for the transmission of acute pain *via* A-fiber afferents.

Selective pain transmission is further supported by the segregation observed in Fos expression between both superficial and deep spinal cord dorsal horns in response to differing pain stimuli. Fos is a nuclear protein produced in cells following expression of the protooncogene c-fos, which is induced by noxious stimuli in neurons that possess the gene that can be immunohistochemically labeled (53, 66).

In rodent models, acute noxious mechanical peripheral stimulation of cutaneous and bone tissue increased Fos expression in the superficial dorsal horn but not in the deep dorsal horn lamina of the spinal cord ipsilateral to the stimulus (53, 56, 67, 68). However, with the application of inflammatory stimuli (similar to cancer-induced pain), the deep dorsal horn lamina becomes most active (66). Similarly, in animal models for cancer-induced bone pain and non-malignant skeletal pain, there is also increased Fos expression in both the superficial and deep dorsal horn (69, 70). However, superficial dorsal horn expression is seen only after the application of additional acute mechanical stimulation (53). While not fully established, the spinothalamic tract and postsynaptic dorsal column pathways likely also play a role in the transmission of inflammatory and/or chronic cancer-induced bone pain, as suggested by the increased Fos expression of the deep dorsal horn (53, 56). This highlights the differences in pain transmission between chronic and acute pain pathways and infers the possibility of top-down hypothalamic modulation of chronic pain from cancer through regional changes in the neuronal activity.

### Hypothalamic Role in Cancer Pain Relief *via* Hypophysectomy

Patients commonly experience temporary episodes of euphoria, polyphagia, hypothermia, and hallucinations following NALP, implicating changes to the hypothalamus as a consequence of the procedure (21). The role of the hypothalamus in pain relief following hypophysectomy was also explored, especially since the role of endorphins and neuroendocrine changes could not wholly explain the mechanism of pain relief.

The hypothalamus is comprised of roughly 40 nuclei. The neurosecretory functions are located mainly in the paraventricular and supraoptic nuclei within the anterior hypothalamus. The secretory functions are subdivided into two neuronal groups: the magnocellular (oxytocin and vasopressin) and parvocellular (corticotropin, gonadotropin, thyrotropin, somatostatin, dopamine, angiotensin II, and growth hormone) neurosecretory cells. These neurons and their secretory products innervate the median eminence and are transported to the anterior pituitary *via* the hypophysial portal system (71).

Postmortem investigations in patients who underwent NALP showed evidence of subependymal gliosis along the floor of the third ventricle, and retrograde degeneration of the supraoptic nuclei, paraventricular nuclei, and median eminence (27). While the extent of anterior hypothalamic damage has not been correlated with duration of pain relief, it possible that this destruction disrupts the regular transport of vasopressin, oxytocin, and other neuroactive peptides (49). Alternatively, anterior hypothalamic degeneration may disrupt the function of neighboring cells that project to downstream pain modulating centers of the brainstem and spinal cord, resulting in antinociception (22, 26, 27, 29, 49).

Rodent studies have demonstrated that intraventricular administration of vasopressin causes dose-dependent pain relief (72). Additionally, vasopressin secretory fibers reach multiple periventricular regions involved with nociception, including the parts of the limbic forebrain, diencephalon, and mesencephalon (29, 73, 74). Oxytocin also has antinociceptive effects. Stimulated paraventricular release of oxytocin produces an endogenous analgesic effect that reduces both Aδ and C-fiber primary afferent signaling in superficial dorsal horn neurons (75, 76).

**Figure 3:**
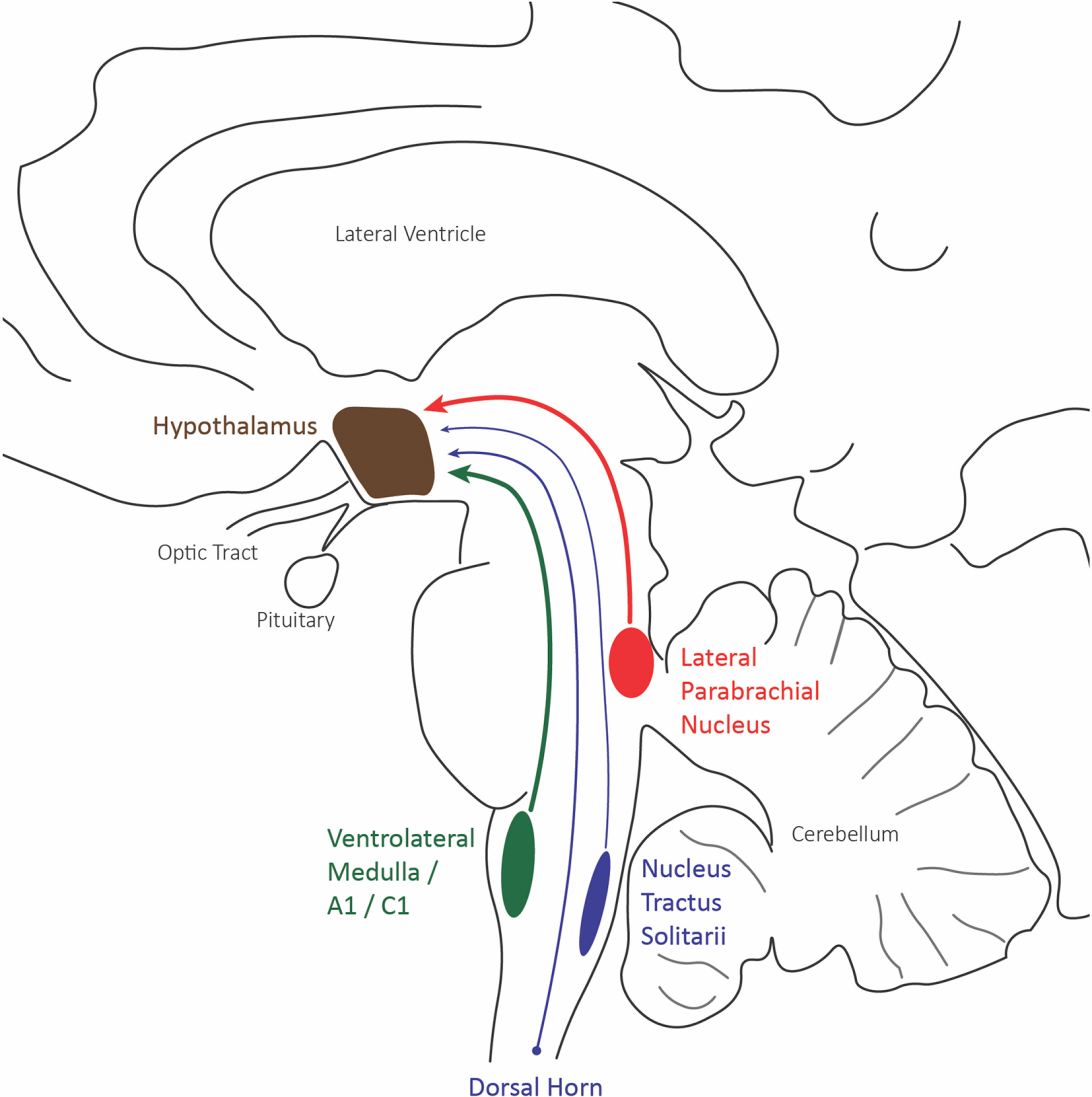
Hypothalamus afferent nociceptive pathways. Sagittal schematic of the three main nociceptive inputs to the hypothalamus: the lateral division of the parabrachial nucleus (red), the caudal ventrolateral medulla including A1/C1 catecholaminergic neurons (green), and the trigeminal and spinal dorsal horn.

Most current evidence for the role of the hypothalamus in nociceptive pain comes from anatomical and c-fos data investigating neural activity. The hypothalamic neurons of the paraventricular, supraoptic, and periventricular nuclei have shown the most robust c-fos expression to evoked painful stimuli. These nuclei all receive nociceptive inputs from the superficial and deep lamina of dorsal horn neurons of the spinal cord, the parabrachial inputs, and caudal ventrolateral medulla inputs, which include the A1/C1 catecholaminergic neurons (**Figure 3**) (71).

**Figure 4:**
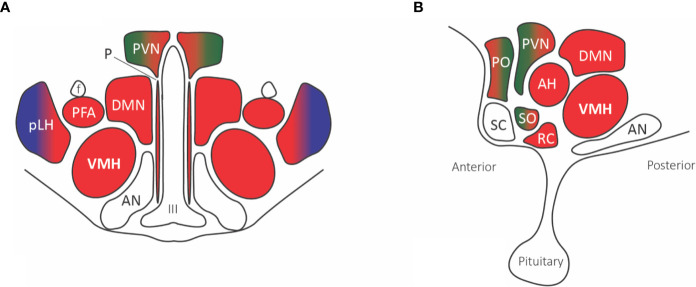
Hypothalamic nuclei with nociceptive projections. **(A)** Coronal view, **(B)** Sagittal view. The predominant parabrachial nociceptive projection (red) is onto the ventromedial hypothalamic nucleus (VMH), with additional projections onto the dorsomedial nucleus (DMN), prefornical area (PFA), periventricular nucleus (PVN), retrochiasmatic area (RC), anterior hypothalamic area (AH), and anterior ventral preoptic nucleus. The parabrachial nucleus (red) and ventrolateral medulla A1/C1 noradrenaline and adrenaline cells (green) project to the paraventricular nucleus (PVN), median preoptic nucleus, and supraoptic nucleus. The posterior part of the lateral hypothalamus (pLH) receives projections from both the parabrachial nucleus and (blue) spinal and trigeminal dorsal horn cells. Abbreviations: AH, anterior hypothalamic area; AN, arcuate nucleus; DMN, dorsomedial nucleus; f, fornix; III, third ventricle; PFA, prefornical area; pLH, posterior lateral hypothalamus; P, periventricular nucleus; PO, preoptic; PVN, paraventricular; RC, retrochiasmatic area; SC, suprachiasmatic nucleus; SO, supraoptic nucleus; VMH, ventromedial hypothalamic nucleus.

The supraoptic nuclei have no known external projections outside of the hypothalamohypophyseal tract. The parabrachial and A1/C1 neurons provide projections to the paraventricular nucleus that innervates magnocellular neurons affecting the secretion of both oxytocin and vasopressin, known to play a role in the visceromotor and neuroendocrine responses to pain (**Figure 4**). Additionally, many paraventricular neurons, along with other hypothalamic areas, send descending projections to the brain stem and spinal cord, affecting the preganglionic sympathetic column responsible for the autonomic response to pain (71).

Hypothalamic stimulation, as opposed to destruction, may account for the differences in adverse events seen with SRS hypophysectomy (8). Evidence to suggest this theory includes: 1) There has been no radiographic evidence of destructive changes, endocrinological dysfunction or morphological changes on follow-up MR imaging; 2) clinical symptoms suggest a hypothalamic stimulatory effect with rapid recovery of appetite and generally improved condition; and lastly 3) MR spectroscopy revealed hypothalamic stimulation with remarkably increased levels of N-acetyl aspartate within 24 hours after radiosurgery, suggestive of increased neuronal activity (8, 39). Regardless, the low rate of adverse events remains promising and may help to revitalize radiosurgery hypophysectomy as a palliative treatment to improve end-stage quality of life for refractory cancer-pain patients with severe pain.

## Conclusion

Stereotactic hypophysectomy has a long history for the palliation of refractory cancer pain. While it has fallen out of common use in recent decades, the minimally invasive and more tolerable radiosurgery hypophysectomy has resurfaced as a possible treatment for cancer-related pain. While the mechanism of pain relief is not entirely understood, the hypothalamohypophyseal axis appears to play an essential role in pain perception and transmission.

Radiosurgery hypophysectomy studies to date have been small, non-randomized, and prospective trials providing low-level evidence. As such, its use should be limited to the palliative treatment of patients with intractable cancer pain that limits the patient’s function and quality of life and is uncontrolled by more traditional treatment options. A multidisciplinary treatment team should thoroughly review patients before consideration of treatment. There have been no comparison studies or SRS hypophysectomy to other pain management options. Further clinical research investigating the use of stereotactic radiosurgery hypophysectomy for refractory pain is currently underway to address several questions, including establishing the difference in pain relief between both hormone and non-hormone responsive malignancy, as well as between malignant and non-malignant pain syndromes. Additionally, optimal radiation dose, targeting, and long-term pain outcomes need further investigation before becoming part of the armamentarium for palliative treatment of cancer-related pain. As additional safety and efficacy data support the use of radiosurgery hypophysectomy, additional radiation modalities may become targets for future research.

Stereotactic hypophysectomy aims to provide relief to populations of patients suffering from medically refractory pain, especially the subpopulation of diffuse metastatic bony pain secondary to hormone-responsive cancer.

## Author Contributions

ML conceived and designed the study, drafted the article, critically revised the article, reviewed the submitted the version of the manuscript, and approved the final version of the manuscript on behalf of all the authors. PK conceived and designed the study, critically revised the article, reviewed the submitted the version of the manuscript, and was in charge of image production. JM acquired the data, critically revised the article, and reviewed the submitted version of the manuscript. IM, and AV critically revised the article, reviewed the submitted version of the manuscript, approved the final version of the manuscript on behalf of all the authors, and supervised the study. All authors contributed to the article and approved the submitted version.

## Conflict of Interest

The authors declare that the research was conducted in the absence of any commercial or financial relationships that could be construed as a potential conflict of interest.
